# Ophthalmology

**Published:** 1898-01

**Authors:** Alvin A. Hubbell

**Affiliations:** Buffalo, N. Y.; Professor of ophthalmology in Niagara University Medical College


### OPHTHALMOLOGY.

CONDUCTED BY ALVIN A. HUBBELL, M. D., Buffalo, N. Y.
Professor of ophthalmology in Niagara University Medical College.

### OPERATION FOR DIVERGENT STRABISMUS.

CHARLES BELL TAYLOR (*Medical Press and Circular*, March 10, 1897,) corrects divergent strabismus by the following method : He hooks up the tendon of the internal rectus muscle and divides all its connections with the eyeball, then pulls it well forward and, by two horizontal incisions made with scissors, converts it into a narrowish strip. He then transfixes the base of this strip as far back in the orbid as possible with a large curved needle connected with a thread, armed at both ends. Before drawing the thread through he cuts off as much of the tendon as may be considered necessary. With the remaining needle he pierces a small portion of the sclerotic about the site of the insertion of the inferior rectus tendon and, having thus secured control of the eye-ball, turns it inward to any required extent by simply tying the thread. The only portion of the procedure which presents any difficulty is the piercing of the sclerotic. To overcome this he uses needles of the same caliber throughout and as sharp as possible and makes the insertion of the thread the first step of the operation.—*American Journal of the Medical Sciences*, August, 1897.

---

### THE ETIOLOGY OF DACRYOCYSTITIS.

Dr. A. A. Foucher, (*Ophthalmic Record*, October, 1897,) of Montreal, at the recent meeting of the British Medical Association, presented a paper upon The Etiology of Dacryocystitis, in which he says : There exist numerous causes of dacryocystitis, but its principal element lies in the unhealthy constitutional state of individuals. This conclusion is arrived at after the study of 273 cases. The adjuvant causes, inflammation of the adjoining parts, change in pathological character of secretion passing through ducts being brought about by debilitating diseases, *i. e.*, variola, tuberculosis, syphilis and the like. Women are more prone to the affection, being of a weaker constitution, are more often ill, pass through critical periods, menstruation, parturition and lachrymation, uterine troubles and menopause, all of which affect the lachrymal ducts more or less, to say nothing of the impressionable nature of women and the very frequent causes which act by reflexion on their lachrymal ducts. Apart from this, the system being prepared by some constitutional unhealthiness or another, interior and exterior infections are favored. Circumstances being now propitious to the production and cultivation of organic microbes and the organism being powerless to render the evil certain, diathesical manifestations appear and among them we classify dacryocystitis.

---

### EUCAINE ANESTHESIA.

Jos. E. Willetts, M. D., (*Medical Review of Reviews*, Nov., 1896,) Pittsburgh, ophthalmologist to the Eye and Ear Hospital of Pittsburgh ; otologist to the Roselia Foundling Asylum, in an address on Ophthalmology, delivered at the meeting of the Medical Society of Pennsylvania, May 19, 1897, says :

"The new local anesthetic, eucaine hydrochlorate, is a decided irritant. The anesthesia of an eucainised eye is accompanied by an hyperemia of the entire conjunctiva which outlasts the anesthesia. Experiments have shown that it also causes a lachrymal hypersecretion. These two factors alone debar it from ophthalmic practice. In corneal ulcers, foreign bodies and the like, the predominant symptoms are photophobia, lachrymation, congestion and pain. The application of this drug relieves the pain, but increases the conditions that produce it. The makers of the drug, when they recommend it in ophthalmic work, ask the ophthalmologist to ignore the fundamental basis of the science of medicine and treat effect instead of cause. Its sole claim to ophthalmic work are its stability and that it does not cause exfoliation of the corneal epithelium. The former ought not to be considered, as the expense incurred for a fresh solution of cocaine hydrochlorate is slight and the latter advantage is not sufficient to cover the other objection.

---

### TREATMENT OF SYMPATHETIC OPHTHALMIA BY THE EXTRACT OF THE CILIARY BODY OF THE OX.

Louis Dor (*Gaz. Hebdomad.*, Ann. 44, No. 50,) has been led by theoretic considerations, including the idea that sympathetic inflammation of the eye results from an altered composition of the intraocular fluids, to try the effect of the instillation into the conjunctival sac of an organic extract prepared by macerating the ciliary body of the eye of the ox. He reports the case of a man who was attacked with sympathetic ophthalmia one year before, and in whom removal of the exciting eye, and subsequent active treatment, including mercurial inunctions and injections, and iridectomy, had failed to prevent blindness, with pain and hyperemia of the eyeball. The regular instillation of the extract of the ciliary body was followed by great improvement in all his symptoms, his improved vision allowing him once more to find his way alone.

In another case of less severe sympathetic disease the improvement under the same treatment was equally noticeable.—*American Journal of the Medical Sciences*, October, 1897.

